# A retrospective comparative study of CT and MRI findings of different types of pancreatic serous cystic neoplasms: Correlation with histopathology

**DOI:** 10.1097/MD.0000000000050307

**Published:** 2026-08-21

**Authors:** Guangmang Li, Aichun Lei, Jianli Zhao, Peng Wang, Guanghai Ji, Xiao Han, Peng Li, Bo Li

**Affiliations:** a Department of Radiology, Huaian Hospital of Huaian City, Huai’an Clinical Medical College of Jiangsu University, Huaian, China; b Department of Trauma Surgery, The Central Hospital of Wuhan, Tongji Medical College, Hua Zong University of Science and Technology, Wuhan, China; c Department of Radiology, The First Affiliated Hospital of Yangtze University, Jingzhou, China; d Department of Pathology, Huaian Hospital of Huaian City, Huai’an Clinical Medical College of Jiangsu University, Huaian, China; e Department of Tumor Therapy, Huaian Hospital of Huaian City, Huai’an Clinical Medical College of Jiangsu University, Huaian, China.

**Keywords:** computed tomography, magnetic resonance imaging, pancreatic neoplasms, serous cystic neoplasm

## Abstract

A comparative analysis of the imaging and pathological findings across different types of serous cystic neoplasms (SCN) was conducted to delineate their distinct imaging characteristics and differentiate between various types of serous cystadenomas. This retrospective analysis included 65 patients diagnosed with SCN, categorized into macrocystic, microcystic, mixed, and solid types based on morphology. Imaging findings were compared across these types to elucidate their characteristics, and microscopic pathological observations were analyzed to identify differences among the SCN types. A total of 65 SCN patients (macrocystic, n = 18; mixed, n = 17; microcystic, n = 29; solid, n = 1) were analyzed. No significant differences were found in age, symptoms, or lesion location among subtypes (*P* > .05), but mixed type lesions were significantly larger than microcystic type (*P* < .05). Computed tomography (CT) and magnetic resonance imaging revealed distinct morphological and enhancement patterns: macrocystic type typically presented as unilocular or paucilocular cysts with no evident enhancement; mixed and microcystic types showed multiple cystic cavities, lobulated contours, fibrous central scars, and varying degrees of enhancement, with significantly higher precontrast CT values than the macrocystic type (*P* < .05). The solid type (n = 1) exhibited a small, round mass with marked arterial enhancement and subsequent washout. Inter-observer agreement for CT quantitative parameters was excellent (intragroup correlation coefficient: 0.966–0.982). Imaging features including central scar, vascular displacement pattern, and margin characteristics differed significantly among subtypes (*P* < .05), supporting the morphological classification of SCN. Light microscopy revealed that macrocystic types had large cyst cavities with thin fibrous septa, while microcystic types exhibited small cavities with dense fibrous septa. Mixed types presented a combination of both characteristics, and solid types showed very small cavities with abundant blood vessels. Imaging findings vary among different subtypes of SCN, and these differences are associated with their pathological features. The imaging characteristics are helpful for noninvasive differentiation of SCN subtypes and guidance of clinical decision-making.

## 1. Introduction

Pancreatic serous cystic neoplasm (SCN) is a relatively uncommon benign tumor, often incidentally detected due to advancements in imaging technology.^[1]^ Although various guidelines propose different management strategies for SCN,^[2-4]^, asymptomatic cases typically do not require surgical intervention. A multicenter study involving 2262 patients reported a low mortality rate of 0.1% associated with SCN, compared to a surgical mortality rate of 0.6%.^[5]^ Despite its benign nature, SCN is sometimes subjected to unnecessary surgical procedures due to misdiagnosis as other tumor types.^[6]^ Hence, accurate diagnosis of SCN is critical, though the wide variability in imaging presentations often leads to a high rate of misdiagnosis.^[1]^

SCN can be classified into 4 morphological types^[7]^: macrocystic, microcystic, mixed, and solid. Microcystic SCN comprises multiple small cysts, each <1 cm in size, separated by thin fibrous septa, sometimes giving a honeycomb appearance. Macrocystic SCN consists of 1 or more cystic lumens measuring 1 cm or more. Mixed-type SCN exhibits features of both macrocystic and microcystic forms, while solid-type SCN lacks visible cystic cavities on cross-sectional imaging. These diverse imaging appearances, ranging from purely cystic to completely solid, present significant diagnostic challenges for radiologists.^[8,9]^

Previous imaging-pathology studies on SCN have largely focused on single subtypes, lacked systematic computed tomography (CT)-magnetic resonance imaging (MRI) comparison, and rarely performed detailed histological correlation. Thus, the imaging spectrum across all 4 subtypes and its pathological basis remain poorly understood. To address these gaps, this study: included all 4 morphological subtypes; performed systematic imaging-pathology correlation; and compared CT and MRI findings for subtype differentiation. Subtype differentiation aids preoperative prediction and may influence surgical strategy: classic subtypes favor conservative management, whereas atypical subtypes warrant more aggressive evaluation. Decisions should remain individualized.

This study retrospectively reviewed 65 surgically confirmed SCN cases and aimed to compare the CT and magnetic resonance imaging MRI features of different pathological subtypes, identify imaging characteristics useful for subtype differentiation, and elucidate the mechanisms underlying imaging differences in correlation with pathological features, thereby deepening the understanding of imaging manifestations of this disease and providing clues for future optimization of diagnostic strategies.

## 2. Materials and methods

This study received approval from the Committee of Huaian Hospital in Huaian City (No. 2024016), and the requirement for informed consent was waived. A retrospective analysis was conducted on patients diagnosed with SCN between March 2017 and July 2023. Lesion classification was based on the 2019 WHO Classification of Tumours of the Digestive System and imaging-based subtyping.^[7,10]^ Inclusion criteria required histological confirmation of SCN via surgical or endoscopic biopsy, completion of MRI or CT plain scans and dynamic contrast-enhanced scans within 30 days prior to surgery, clear visibility of the lesion on imaging, and no prior treatment before the examination. Demographic data (age, sex), clinical information (symptoms, tumor markers), imaging results (CT and MRI), and pathological data were collected for comparative analysis.

### 2.1. Image inspection parameter setting and data acquisition

Multidetector CT (Philips Brilliance iCT; GE Discovery CT750; GE LightSpeed VCT) was performed for unenhanced upper abdominal and dual-phase contrast-enhanced pancreatic scanning. The scan range covered from the diaphragm dome to the lower margin of the kidneys. The tube voltage was set at 120 kV, with a slice thickness of 5 mm. A total of 1.5 mL/kg of iohexol (350 mgI/mL) was injected via the antecubital vein at a rate of 3 mL/s, and images were acquired during the arterial phase (35 seconds) and portal venous phase (65 seconds).

MRI was performed using GE Signa Explorer 1.5T and GE Signa Architect 3.0T scanners. The protocol included axial and coronal fat-suppressed T1-weighted imaging (T1WI), fat-suppressed T2-weighted imaging (T2WI), and LAVA dynamic contrast-enhanced imaging. For dynamic contrast-enhanced imaging, gadopentetate dimeglumine (0.1 mmol/kg) was injected at a rate of 2.0 mL/s, and dynamic scanning began 15 seconds after the start of contrast injection.

### 2.2. Image analysis and measurement

CT and MR images for all patients were imported into the picture archiving and communication system system for evaluation. Two radiologists, with 10 and 8 years of experience in imaging diagnosis respectively, independently conducted the evaluation in a blinded manner with regard to the clinical and pathological results. Quantitative parameters were measured independently by both radiologists. In cases of any inconsistency in qualitative assessment, a senior radiologist participated in the evaluation. The quantitative parameters measured by both physicians were then tested for consistency.

The qualitative parameters assessed from CT images included tumor location, shape, boundary, intracranial calcification, fibrous central scar, pancreatic duct dilation, the relationship between lesions and peripancreatic blood vessels (categorized as no relationship, extrusion, blood supply, invasion), peripancreatic lymph node enlargement (defined as a lymph node diameter ≥ 1.0 cm), liver metastasis, and distant metastasis. Additionally, the lesion density in precontrast scans, arterial phase, and portal phase were evaluated using the pancreatic parenchyma as a reference.

The maximum long diameter and the maximum short diameter perpendicular to it were measured on the largest 2-dimensional cross-sectional image. Tumor size was calculated as the average of these 2 measurements. Quantitative CT parameters included lesion size, precontrast CT value of the lesion and surrounding normal pancreatic parenchyma (CTun), enhanced arterial phase CT value (CTap), and portal phase CT value (CTpp). The largest dimension of the tumor was selected for lesion size measurement. Lesion length and diameter were measured 3 times, and the average was taken. When measuring CT values on contrast-enhanced images, the region of interest (ROI) was placed in the area of greatest enhancement within the solid component, avoiding cystic, necrotic, calcified, or vascular areas. The ROI covered 1/2 to 2/3 of the solid area, and measurements were repeated 3 times to obtain an average. For multilocular cystic tumors without an obvious solid component, the smallest feasible ROI was placed on the thickest portion of the cyst wall or septum. Absolute enhancement values for the arterial phase were calculated as ΔCTap = CTap − CTun, and absolute enhancement values for the portal phase were calculated as ΔCTpp = CTpp − CTun. The arterial phase relative enhancement index was calculated as the enhanced value in the arterial phase divided by the enhanced value in the surrounding pancreas. The portal vein phase relative enhancement index was calculated similarly. The degree of enhancement was categorized as no enhancement (<10 HU), mild enhancement (10–30 HU), moderate enhancement (30–50 HU), and significant enhancement (>50 HU).

MR images were assessed for lesion size, location, shape, boundary, T1WI and T2WI signal intensity (relative to hepatic parenchyma), fibrous central scar, pancreatic duct dilation, and the relationship between the lesion and pancreatic duct as observed on lipid-pressure sequence. Additionally, the relationship between the lesion and surrounding structures and the presence of metastasis were evaluated.

### 2.3. Pathological examination method

Tumor tissues were fixed using a 4% neutral formaldehyde solution, followed by paraffin embedding, with sections cut to a thickness of 4 μm and stained with hematoxylin and eosin. Morphology and cytoplasmic staining of tumor cells were observed under an optical microscope. The tumors were grouped according to different imaging classifications and compared with their respective imaging findings.

All pathological specimens (surgical resections and endoscopic ultrasound-guided fine-needle aspiration biopsies) were reviewed by 2 experienced gastrointestinal pathologists (with > 10 years of specialty experience), who were blinded to the imaging findings. The diagnosis of SCN was established according to the 2019 WHO Classification of Digestive System Tumors, based on the characteristic histomorphological features: cystic spaces lined by cuboidal or flattened epithelial cells with clear glycogen-rich cytoplasm; periodic acid-Schiff-positive, diastase-labile cytoplasmic staining; and immunohistochemical positivity for cytokeratin (CK7, CK19) and negative staining for mucin markers (MUC1, MUC2, MUC5AC). For biopsy-confirmed cases, diagnostic certainty was established by: the presence of diagnostic histomorphological features on adequate tissue cores obtained by fine-needle aspiration biopsy; confirmatory histochemical and immunohistochemical staining as described above; and consensus agreement between the 2 reviewing pathologists. Cases with discordant interpretations or insufficient material were excluded.

### 2.4. Statistical method

SPSS 23.0 (IBM Corporation, Chicago) was used for data analysis. Qualitative variables are expressed as frequencies (%). Measurement data were tested for normality and homogeneity of variance, and normally distributed data were expressed as mean ± standard deviation. ANOVA was used for multi-group comparisons of normally distributed data. Non-normally distributed data are expressed as median (quartile) and analyzed using nonparametric tests. Qualitative data were compared using the χ^2^ test or Fisher’s exact test. A *P*-value of <.05 was considered statistically significant. The intragroup correlation coefficient (ICC) was used to assess the consistency of quantitative data measured by the 2 physicians. An ICC > 0.9 indicated good consistency.

## 3. Result

### 3.1. General data of different SCN types

A total of 65 patients were included, with 53 undergoing CT and 48 undergoing MRI. Of these, 38 cases were confirmed pathologically after surgery, and 27 cases were confirmed through endoscopic fine-needle aspiration biopsy. A total of 65 patients diagnosed with SCN were analyzed, comprising 18 cases of macrocystic type, 17 cases of mixed type, 29 cases of microcystic type, and 1 case of solid type. The patients’ general characteristics are summarized in Table 1. No statistically significant differences were observed in age, lesion location, clinical symptoms, or underlying diseases among the different SCN types (*P* > .05). However, the lesion volume of the mixed type was significantly larger than that of the microcystic type (*P* < .05), though no significant differences were found between the macrocystic type and the mixed or microcystic types (*P* > .05).

**Table 1:** Comparison of clinical information on different types of SCN.

	Macrocystic type (n = 18)	Mixed type (n = 17)	Microcapsule type (n = 29)	Solid type (n = 1)	*P*	Total (n = 65)
Age (r)	54.6 ± 15.3	60.1 ± 9.93	58.±12.1	62	.432	57.7 ± 12.7
Gender, n (%)						
Male (example)	6 (33.3)	4 (23.5)	9 (31.0)	1	.830	20 (30.8)
Female (example)	12 (66.7)	13 (76.5)	20 (69.0)	0	.830	45 (69.2)
Initial detection size (mm)	34.3 ± 13.9	42.9 ± 15.1	29.6 ± 10.8	12.5	.019	34.1 ± 14.2
Location, n (%)						
Head/neck	6 (33.3)	8 (47.1)	9 (31.0)	0	.744	21 (32.3)
Body/tail	12 (66.7)	9 (52.9)	20 (69.0)	1	.744	44 (67.7)
Clinical symptoms, n (%)						
Abdominal pain	7 (38.9)	3 (17.6)	8 (30.7)	0	.381	18 (27.7)
Physical examination or occasional	11 (61.1)	14 (82.4)	18 (69.3)	1	.320	44 (67.7)
Hyperglycemia	0 (0)	0 (0)	2 (6.9)	0	.497	2 (3.1)
Nausea and vomiting	0 (0)	0 (0)	1 (3.1)	0	1.000	1 (1.5)
CA199, n (%)	1 (5.6)	1 (5.9)	0 (0)	0	.295	2 (3.1)
Basic diseases, n (%)						
Hypertension	6 (33.3)	2 (11.8)	5 (17.2)	0	.314	13 (20.0)
Diabetes	1 (5.6)	0 (0)	2 (6.9)	0	.787	3 (4.6)
Elevated uric acid	1 (5.6)	0 (0)	1 (3.1)	0	1.000	2 (3.1)
Cardiac insufficiency	0 (0)	0 (0)	1 (3.1)	0	1.000	1 (1.5)

The solid type was not included in the statistical difference analysis. SCN = serous cystic neoplasm.

### 3.2. Consistency of CT quantitative parameter measurements

The consistency of the CT quantitative parameters measured by the 2 physicians was evaluated. The ICC values for plain precontrast CT value, arterial phase absolute enhancement value, venous phase absolute enhancement value, arterial phase enhancement index, and venous phase enhancement index were 0.979, 0.966, 0.975, 0.982, and 0.967, respectively, indicating excellent agreement between the 2 observers.

### 3.3. CT findings and comparison of different types of SCN

A total of 53 SCN patients underwent CT examination, with findings and comparative analysis presented in Table 2. Regarding quantitative parameters, mixed and microcystic types showed significantly higher precontrast CT values than the macrocystic type (*P* < .05). The macrocystic type demonstrated no evident enhancement in either arterial or portal phases, significantly differing from the mixed and microcystic types (*P* < .05). No significant differences were observed between mixed and microcystic types in any enhancement parameters (*P* > .05). Regarding morphological features, lobulated contours were seen in 66% of all cases, with the highest proportion in the mixed type (81.8%). Ill-defined margins were observed only in mixed and microcystic types, whereas all macrocystic cases had well-defined margins (*P* < .05). Central fibrous scars were present in mixed and microcystic types but absent in the macrocystic type (*P* < .05). No significant difference in the calcification proportion was found among the 3 types (*P* > .05). Regarding vascular relationships, vessel displacement was predominant in the macrocystic type, while vessel thickening was more common in mixed and microcystic types (*P* < .05).

**Table 2:** Comparison of CT imaging findings of different types of SCN.

Imaging findings	Macrocystic type (n = 17)	Mixed type (n = 11)	Microcapsule type (n = 24)	Solid type (n = 1)	*P*	Total (n = 53)
Precontrast scan CT value (HU)	8 (3.75, 10.25)	24 (14, 27)	18.5 (15, 23)	25	.000	15 (10,23)
Arterial phase absolute enhanced value (HU)	–	47 (30.5, 57)	34 (19.75, 47.25)	81	.122	–
Portal vein phase absolute enhanced value (HU)	–	50 (34.5, 54)	38.5 (26.5, 57)	40	.657	–
API	–	0.792 (0.576, 1.048)	0.544 (0.429, 0.672)	1.333	.073	–
PPI	–	0.770 (0.461, 0.965)	0.612 (0.540, 0.838)	0.923	.722	–
Enhancement degree (arterial phase), n (%)						
No enhancement	17 (100.0)	0 (0)	0 (0)	0	.000	17 (32.1)
Mild enhancement	0 (0)	3 (27.3)	7 (29.2)	0	.029	10 (18.9)
Moderate enhancement	0 (0)	2 (18.2)	9 (37.5)	0	.008	11 (20.7)
Obvious enhancement	0 (0)	6 (54.5)	8 (33.3)	1	.002	15 (28.3)
Enhancement degree (portal vein phase), n (%)						
No enhancement	17 (100.0)	0 (0)	1 (4.2)	0	.000	18 (34.0)
Mild enhancement	0 (0)	3 (27.3)	10 (41.7)	0	.004	13 (24.5)
Moderate enhancement	0 (0)	3 (27.3)	8 (33.3)	1	.018	12 (22.6)
Obvious enhancement	0 (0)	5 (45.6)	5 (20.8)	0	.007	10 (18.9)
External contour, n (%)						
Smooth	4 (23.5)	0 (0)	5 (20.8)	1	.281	10 (18.9)
Lobulated	12 (70.6)	9 (81.8)	14 (58.6)	0	.373	35 (66.0)
Ambiguity	1 (5.9)	2 (18.2)	5 (20.8)	0	.473	8 (15.1)
Boundary, n (%)						
Clear	17 (100.0)	8 (72.7)	16 (66.7)	1	.018	42 (79.2)
Unclear	0 (0)	3 (27.3)	8 (33.3)	0	.018	11 (20.8)
Fibrous central scar, n (%)	0 (0)	4 (36.4)	3 (12.5)	0	.021	7 (13.2)
Internal calcification, n (%)	3 (17.6)	4 (36.4)	8 (33.3)	0	.488	15 (28.3)
Pancreatic duct dilation, n (%)	1 (5.9)	3 (27.3)	4 (16.7)	1	.331	9 (17.0)
Relationship with blood vessels, n (%)						
No relationship	4 (23.5)	0 (0)	1 (4.2)	0	.097	5 (9.4)
Squeeze	12 (70.6)	3 (27.3)	5 (20.8)	0	.004	20 (37.7)
Blood-supply	1 (5.9)	8 (72.7)	18 (75.0)	1	.000	28 (52.9)
Distant organ and lymph node metastasis, n (%)	0 (0)	0 (0)	0 (0)	0	NS	0 (0)

The solid type was not included in the statistical difference analysis.

API = arterial phase enhancement index, PPI = portal vein phase enhancement inde, SCN = serous cystic neoplasm.

**Figure 1.**
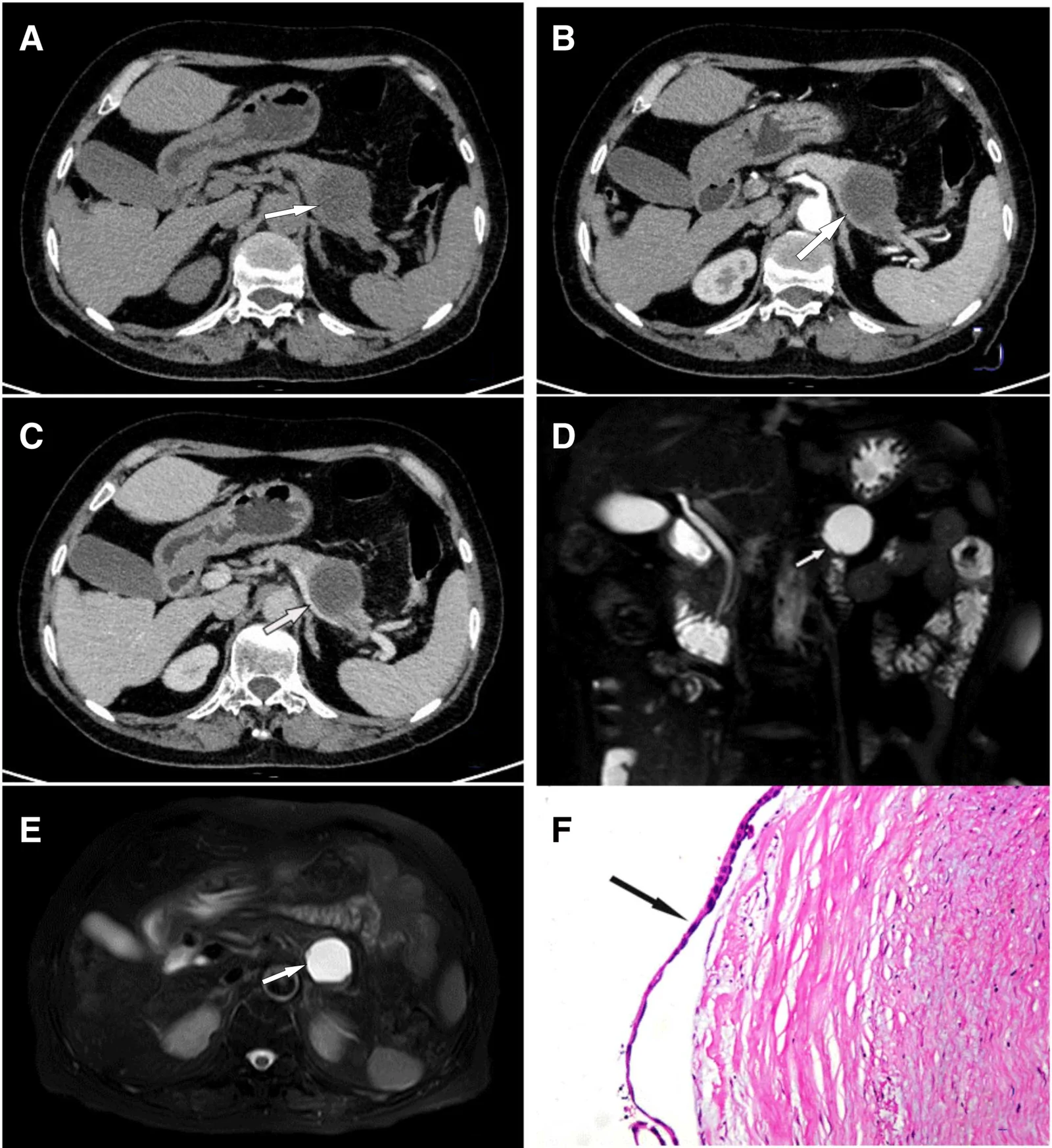
CT and MR images of a 68-year-old female patient with SCN (macrocystic type) in the tail of the pancreas. (A) Precontrast axial CT scan showing a round, low-density cystic lesion (arrow) in the tail of the pancreas. (B) Arterial-phase CT enhancement reveals a cystic lesion (arrow) in the tail of the pancreas, with clear lesion edges and no obvious enhancement. (C) Portal vein phase CT enhancement shows no significant enhancement in the pancreatic tail lesion, which compresses the posterior splenic vein without vascular invasion (arrow). (D) Axial T2WI lipid-pressure sequence shows a circular high-signal lesion (arrow) in the tail of the pancreas with clear margins. (E) Coronal T2WI lipid-pressure sequence shows a high-signal lesion in the pancreatic tail (arrow) with a thin internal septum visible in the lower part. (f) HE staining at ×200 magnification shows only the capsule wall, which is thin (arrow). The capsule wall is lined with a single layer of cuboidal epithelial cells and thin fibrous collagen underneath. Fibrous tissue outside the capsule wall shows signs of hyperplasia. CT = computed tomography, HE = hematoxylin and eosin, MR = magnetic resonance, SCN = serous cystic neoplasm, T2WI = T2-weighted images.

**Figure 2.**
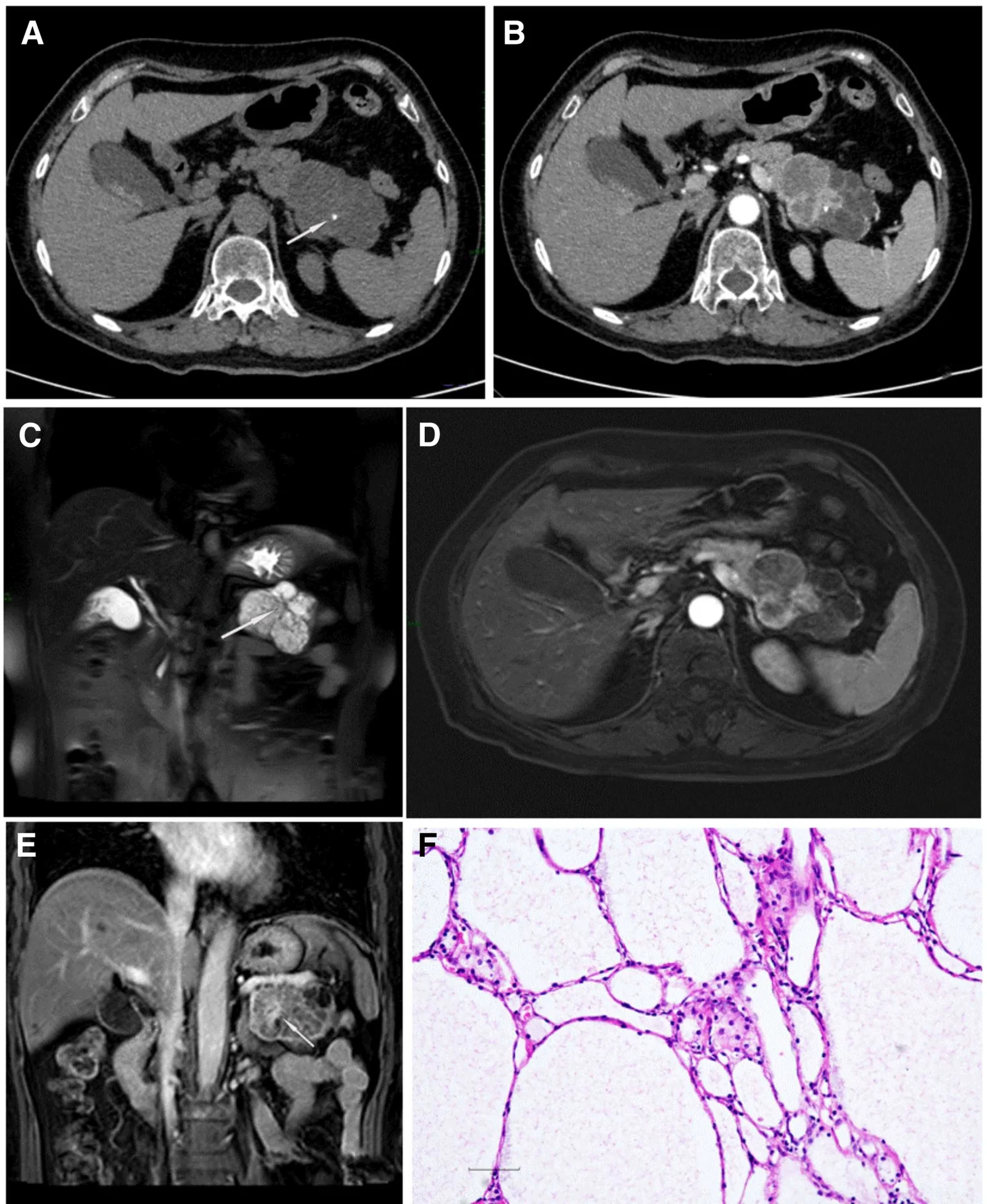
CT and MR Images of a 52-year-old female patient with SCN (mixed type) in the tail of the pancreas. (A) Axial CT precontrast scan shows lobulated outer margins with punctate calcifications inside (arrow). (B) Arterial-phase CT enhancement reveals clear lesion edges, uneven internal enhancement, and obvious parenchymal and cyst wall enhancement (arrow), but no enhancement in the cystic cavity. (C) Coronal T2WI lipid-pressure sequence shows high-signal lesions in the pancreatic tail, with clear edges, multiple lobulated outer margins, and visible low-signal septa and fibrous scars (arrow) inside. (D) Axial MRI reveals uneven enhancement of the lesion (arrow), with significant enhancement of the dense microcystic areas and cyst wall, but no enhancement in the larger cyst cavity. (E) Coronal MRI enhancement shows multiple compartmented enhancements and delayed scar enhancement (arrow) inside the lesion, with varying capsule sizes and no enhancement within the cystic cavities. (F) HE staining at ×200 magnification reveals capsules of different sizes lined with a single layer of flat (arrow) or cuboidal epithelial cells, with abundant fibrous tissue inside the tumor. Small cystic cavities and small blood vessel proliferation are visible within the fibrous septa. CT = computed tomography, HE = hematoxylin and eosin, MRI = magnetic resonance imaging, SCN = serous cystic neoplasm, T2WI = T2-weighted images.

**Figure 3.**
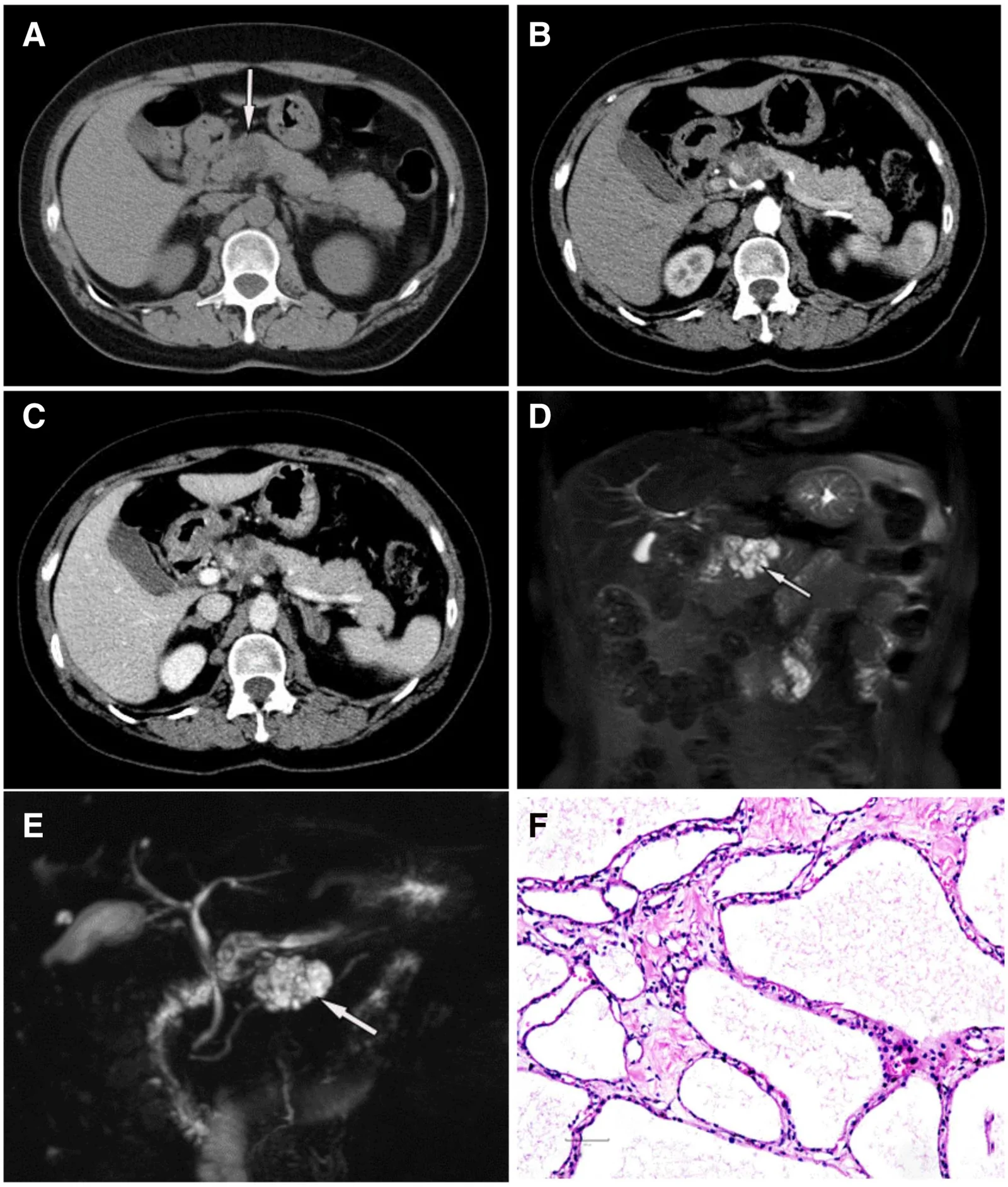
CT and MR images of a 58-year-old male patient with SCN (microcystic type) in the head of the pancreas. (A) Axial CT precontrast scan shows a cystic mass in the head of the pancreas (arrow). (B) Arterial-phase CT enhancement shows an irregularly shaped pancreatic head mass (arrow) with uneven enhancement and small patches of significant enhancement. (C) Portal vein phase CT enhancement shows nonuniform enhancement of the pancreatic head lesions (arrow) with no signs of vascular invasion. (D) Coronal T2WI lipid-pressure sequence shows high-signal lesions in the head of the pancreas, with multiple lobulated profiles (arrows), clear edges, and visible low-signal compartments and fibrous scars inside. (E) 3D-lipid-pressure sequence shows high-signal lesions in the pancreatic head with multiple microsacs and a lobulated external contour (arrow), with no dilation of the pancreatic duct. (F) HE staining at ×200 magnification shows that the lesion is composed of numerous small sacs of varying sizes. The fibrous interstitium is relatively abundant, with smaller cystic cavities and small vascular structures visible within the fibrous septa and interstitium (arrow). CT = computed tomography, HE = hematoxylin and eosin, MR = magnetic resonance, SCN = serous cystic neoplasm, T2WI = T2-weighted images.

**Figure 4.**
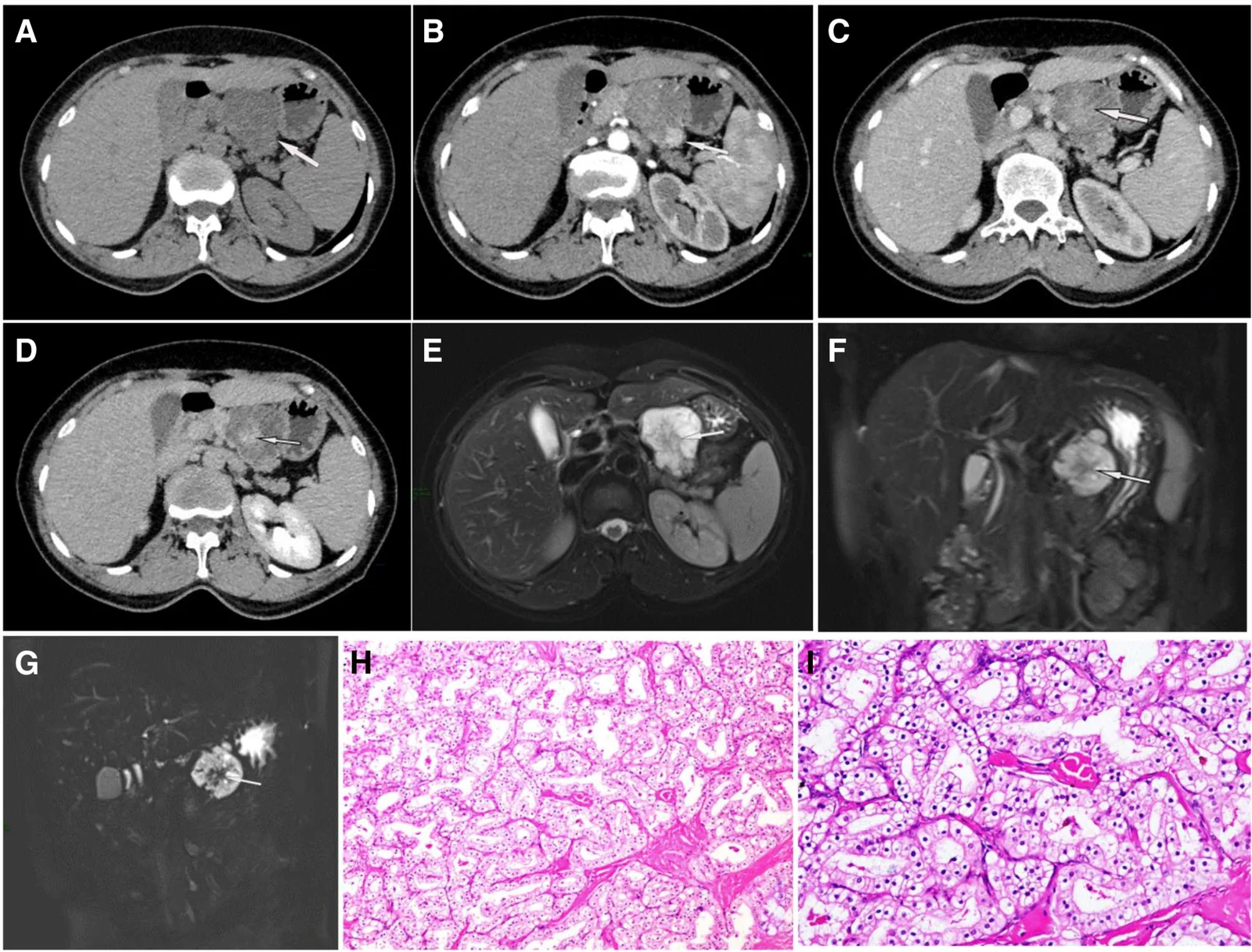
CT and MR images of a 63-year-old female patient with SCN (microcystic type) in the tail of the pancreas. (A) Axial CT precontrast scan shows a lesion in the pancreatic tail (arrow) with clear boundaries. (B) Arterial-phase CT enhancement reveals uneven enhancement, with obvious enhancement foci (arrow) at the lesion’s edges. (C, D) Portal vein phase and delayed phase CT enhancement show delayed enhancement of the internal fibrous scar (arrow). (E) Axial T2WI lipid-pressure sequence shows high-signal lesions in the pancreatic tail with clear edges, a lobulated contour, multiple internal separations, and central fibrous scars (arrow). (F) Coronal T2WI lipid-pressure sequence reveals small cystic external protrusions on the lesion’s outer edge, showing the “extracapsular sac” sign, with a visible isolow-signal fibrous scar in the center (arrow). (G) 2D-lipid-pressure sequence shows a lesion with obvious hypersignal, and the interior clearly reveals a stellate fibrous scar (arrow). (H) HE staining at ×100 magnification shows multiple microcystics (arrow) structures composed of fibrous septa lined with cuboidal epithelium, fibrous scar tissue, and relatively dense fibrous interstitium. (I) HE staining at ×200 magnification shows hyaloid degeneration and multiple small blood vessels within the fibrous interstitium (arrow). CT = computed tomography, HE = hematoxylin and eosin, MR = magnetic resonance, SCN = serous cystic neoplasm, T2WI = T2-weighted images.

**Figure 5.**
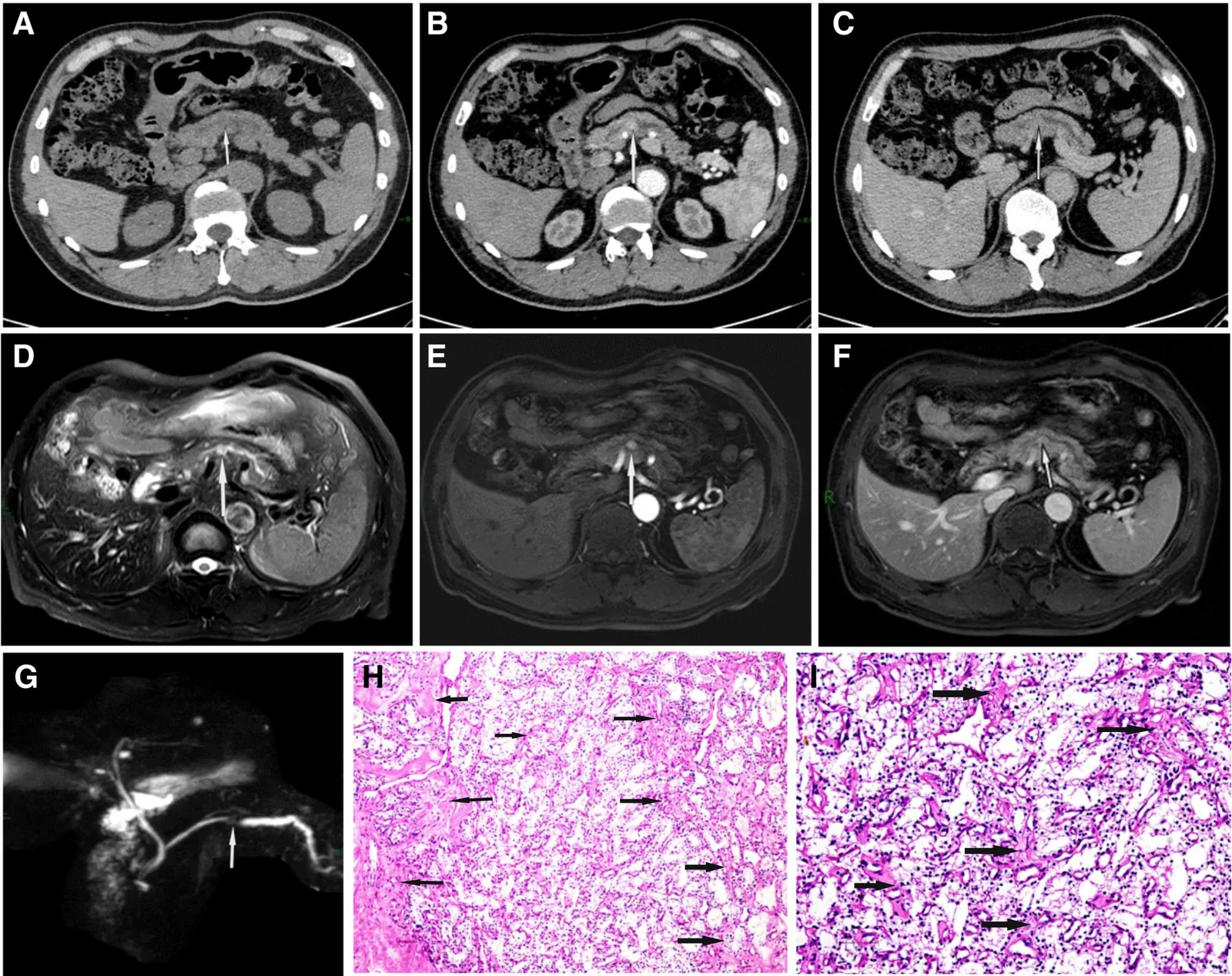
CT and MR images of a 57-year-old female patient with SCN (solid type) in the pancreatic body. (A) Axial CT precontrast scan shows slightly low-density lesions in the pancreatic body (arrow) with unclear boundaries. (B) Arterial-phase CT enhancement shows significant enhancement of the small circular lesion (arrow), with the degree of enhancement higher than that of the pancreatic parenchyma. The lesion is adjacent to the pancreatic duct, which is dilated. (C) Delayed CT enhancement shows that the lesion’s enhancement is similar to that of the pancreas (arrow). (D) Axial T2WI lipid-pressure sequence shows a high-signal lesion (arrow) adjacent to the pancreatic duct. (E) Arterial-phase MR enhancement shows a small circular lesion in the pancreatic body with obvious enhancement (arrow) and compression of the adjacent pancreatic duct. (F) MR enhancement in the portal vein phase shows that the degree of enhancement of the lesion in the pancreatic body (arrow) is similar to that of the pancreatic parenchyma, with pressure changes in the pancreatic duct. (G) 3D-lipid-pressure sequence shows localized pancreatic duct stenosis (arrow) and distal pancreatic duct dilation. (H) HE staining at ×100 magnification shows closely arranged microscopic capsular structures, abundant fibrous interstitium (arrow), and thick fibrous septation. (I) HE staining at ×200 magnification shows cuboidal epithelial cells in the microcystic arrangement, with abundant collagen fibers and interstitium (arrow), and rich in mature small blood vessels. As this is a single case of the solid type, the imaging manifestations should not be considered representative of all solid-type SCNs. CT = computed tomography, HE = hematoxylin and eosin, MR = magnetic resonance, SCN = serous cystic neoplasm, T2WI = T2-weighted images.

Typical findings by subtype were as follows: the macrocystic type (Fig. 1A–C) presented as single or multiple large cysts, with 70.6% showing a lobulated contour, watery density on precontrast scans, and no enhancement; the mixed type (Fig. 2A, B) consisted of cysts of varying sizes, with 81.8% showing a lobulated contour, arterial enhancement of solid areas with delayed scar enhancement; the microcystic type (Figs. 3A–C and 4A–D) consisted of multiple microcysts, with 58.6% showing a lobulated contour, density between fluid and pancreatic parenchyma, and marked arterial enhancement of peripheral solid areas; in our single case of solid-type SCN (Fig. 5A–C), the tumor was small and round, showing marked arterial enhancement with portal venous washout.

### 3.4. MRI findings and comparison of different SCN types

A complete MRI examination was conducted on 48 patients with SCN, and the MRI findings and analysis of various SCN types are outlined below (Table 3).

**Table 3:** Comparison of MRI imaging findings of different types of SCN.

Imaging findings	Macrocystic type (n = 9)	Mixed type (n = 16)	Microcapsule type (n = 22)	Solid type (n =1 )	*P*	Total (n = 48)
T1WI signal strength, n (%)						
High signal	0 (0)	0 (0)	0 (0)	0	NS	0 (0)
Low signal	9 (100.0)	16 (100.0)	22 (100.0)	1	NS	48 (100.0)
T2WI signal strength, n (%)						
High signal	9 (100.0)	16 (100.0)	22 (100.0)	1	NS	48 (100.0)
Low signal	0 (0)	0 (0)	0 (0)	0	NS	0 (0)
External contour, n (%)						
Smooth	2 (22.2)	1 (6.3)	3 (13.6)	1	.546	7 (14.6)
Lobulated	7 (77.8)	15 (93.7)	19 (86.4)	0	.546	41 (85.4)
Boundary, n (%)						
Clear	9 (100.0)	16 (100.0)	22 (100.0)	1	NS	48 (100.0)
Unclear	0 (0)	0 (0)	0 (0)	0	NS	0 (0)
Internal calcification, n (%)	0 (0)	12 (75.0)	12 (54.5)	0	.001	24 (50.0)
Pancreatic duct dilation, n (%)	1 (11.1)	3 (18.7)	3 (13.6)	1	.874	8 (16.7)
Relationship with pancreatic duct						
No relationship	8 (88.9)	13 (81.3)	19 (86.4)	0	.874	40 (83.3)
Squeeze	1 (11.1)	3 (18.7)	3 (13.6)	1	.874	8 (26.7)
Communicating	0 (0)	0 (0)	0 (0)	0	NS	0 (0)

The solid type was not included in the statistical difference analysis.

SCN = serous cystic neoplasm, T1WI = T1-weighted imaging, T2WI = T2-weighted imaging.

All 48 patients exhibited a low signal on T1WI and a high signal on T2WI, with distinct boundaries and no evidence of communication with the pancreatic duct. The majority displayed a lobular contour. No statistically significant differences were observed among the macrocystic, mixed, and microcystic types (*P* > .05). However, a higher incidence of stellate fibrous scars was noted in the mixed and microcystic types compared to the absence of scars in the macrocystic type, and this difference was statistically significant (*P* < .05). No statistically significant differences were found between the mixed and microcystic types (*P* > .05).

Macrocystic type (Fig. 1D, E) showed low T1WI and high T2WI signal, 77.8% lobulated, no enhancement; mixed type (Fig. 2C–E) showed high T2WI signal, 93.7% lobulated, enhancement in small cystic areas with delayed scar enhancement; microcystic type (Figs. 3D, E and 4E–G) showed high T2WI signal, 86.4% lobulated, with internal low-signal fibrous septa/scars. In our single case of solid-type SCN (Fig. 5D–G), the tumor showed high T2WI signal, round shape, arterial enhancement with portal venous washout, and was accompanied by pancreatic duct dilation.

### 3.5. Microscopic pathology of different types of SCN

Under the microscope, sections stained with hematoxylin and eosin revealed that all tumors exhibited cystic structures with a pale red stain. Pathologically, all cases exhibited cystic structures with a single layer of cuboidal epithelial cells lining the inner wall. The different types of SCN displayed variations in cystic structures in terms of size, number, arrangement, thickness of fibrous septa, presence of fibrous scars, proportion of fibrous stroma, and the abundance of small blood vessels.

#### 3.5.1. Macrocystic type

Due to the large cystic cavities, only the capsule wall (Fig. 1F) or fibrous septum is visible under the microscope, with a thin capsule wall or septum. The capsule wall is lined with a single layer of cuboidal epithelial cells and has thin fibrous collagen beneath it. The lining typically favors cuboidal epithelium over flat epithelium. The nuclei are relatively large, the cytoplasm is transparent, and small cysts may occasionally be visible on the wall of a single cyst under the microscope.

#### 3.5.2. Mixed type

The size of the capsules varies (Fig. 2F), and microscopic features show a great deal of variation in different areas, including both large and microcystic manifestations.

#### 3.5.3. Microcystic type

Under the microscope, the incised surface appears spongy and consists of numerous small sacs (Fig. 3F), with diameters mostly ranging from a few hundred micrometers to a few millimeters. The sac walls are lined with a single layer of flat or cuboidal epithelial cells, with bright red cytoplasm, centrally located round-to-oval nuclei, consistent in size, with inconspicuous nucleoli, lacking nuclear division, and showing no atypical cell shapes. The tumor’s fibrous tissue is relatively abundant, with common fibrous scars (Fig. 4H, I). Tiny cystic cavities and small blood vessel hyperplasia are visible under the microscope in the fibrous septum. The smaller the cystic cavity, the more abundant the fibrous stroma and small blood vessels. Fibrous scars consist of relatively dense fibrous stroma, with hyalinoid tissue visible, and small clusters of sacs observed within and around the fibrous scars. Unlike true scar tissue, these fibrous collagens are rich in small vascular structures.

#### 3.5.4. Solid type

Under light microscopy, a case of solid-type SCN demonstrated that very small cavities are visible (Fig. 5H, I), often ranging from tens to hundreds of micrometers, lined with typical cuboidal epithelial cells. These cells are closely arranged in nests, sheets, and trabeculae, separated by thick fibrous bands rich in interstitial components and small blood vessels. These vessels are mature, and the vascular wall structure remains intact.

### 3.6. Value of CT and MRI features in differentiating SCN subtypes

CT and MRI demonstrated characteristic features for each SCN subtype. The macrocystic type showed unilocular or paucilocular large cysts, watery low density on precontrast scans, no significant enhancement, no central scar, and vessel displacement. The mixed type showed coexistence of macro- and microcysts, higher precontrast CT values than the macrocystic type (*P* < .05), variable enhancement, central scars, and vascular thickening/tortuosity. The microcystic type showed multiple microcysts, higher precontrast CT values than the macrocystic type (*P* < .05), variable enhancement, and frequent central scars. A case of solid-type SCN presented as a small solid mass with marked arterial enhancement and washout. T1WI/T2WI signal intensities cannot reliably distinguish SCN subtypes; however, morphological features (e.g., fibrous scars, microcystic architecture, septal enhancement) are useful for subtype differentiation.

To better illustrate the imaging-pathology correlations for each SCN subtype, we summarized the key imaging features and their corresponding histopathological basis in Table 4.

**Table 4:** Imaging-pathology correlation of SCN subtypes.

Subtype	Imaging features	Histopathological basis
Macrocystic	Unilocular or paucilocular large cysts; watery low density on precontrast CT; no significant enhancement; no central scar; vessel displacement	Large cystic spaces lined by cuboidal epithelium; sparse stromal component; few blood vessels
Mixed	Coexistence of macro- and microcysts; higher precontrast CT values than macrocystic type; variable enhancement; central scar; vascular thickening and tortuosity	Variable proportions of cystic spaces and solid stroma; fibrous scar with hyalinization; prominent stromal vasculature
Microcystic	Multiple microcysts with honeycomb appearance; higher precontrast CT values than macrocystic type; variable enhancement; central stellate scar	Densely packed microcysts with abundant fibrotic stroma; rich capillary network; central stellate scar
Solid	Small round solid mass; marked arterial enhancement with portal venous washout; high T2WI signal	Dense cellular stroma with mature small blood vessels; minimal or no cystic component; intact vascular endothelium

Findings for the solid type are derived from a single case and await confirmation in larger studies.

CT = computed tomography, SCN = serous cystic neoplasm, T2WI = T2-weighted images.

### 3.7. CT and MRI misdiagnosis of different types of SCN

Among the 53 patients with SCN who underwent CT examination, 22 (41.5%) were misdiagnosed. The macrocystic type is prone to misdiagnosis as a mucinous cystic neoplasm and other cystic lesions. The mixed type may be mistaken for a neuroendocrine tumor (PanNET). It is crucial to differentiate the microcystic type from various pancreatic cystic-solid tumors. A case of solid-type SCN was misdiagnosed as PanNET. The misdiagnosis rates for different types of SCN on CT are detailed in Table S1, Supplemental Digital Content 1.

Of the 48 patients who underwent MR examination, 15 (31.3%) were misdiagnosed. The macrocystic type is commonly misidentified as MCN, while the mixed, microcystic, and solid types are frequently misdiagnosed as PanNETs. The misdiagnosis rates for different types of SCN on MRI imaging are outlined in Table S2, Supplemental Digital Content 2.

Among misdiagnosed cases, the hypervascular enhancement pattern (mixed and solid types) and unilocular large cystic appearance (macrocystic type) were the most frequent contributors to diagnostic errors. Central scars were observed only in correctly diagnosed cases and were not present in any misdiagnosed case. Lesions misdiagnosed as PanNET were predominantly small (<2 cm) with a pure solid appearance on CT.

## 4. Discussion

This study aimed to compare the CT and MRI features of different pathological subtypes of pancreatic serous cystic neoplasm and to identify imaging characteristics useful for subtype differentiation. Based on a retrospective analysis of 65 patients with surgically or biopsy-proven SCN, we found that mixed and microcystic types showed significantly higher precontrast CT values than the macrocystic type. Central fibrous scars were present only in mixed and microcystic types, while the macrocystic type demonstrated no significant enhancement. A case of solid-type SCN showed marked arterial enhancement with portal venous washout, leading to its misdiagnosis as a pancreatic neuroendocrine tumor. These imaging differences are closely associated with the underlying pathological features of each subtype. Accurate recognition of these features is of great clinical importance for improving preoperative noninvasive differentiation of SCN subtypes, guiding individualized treatment decisions, and avoiding unnecessary surgical interventions.^[4,11]^

Pancreatic SCN is a rare exocrine gland tumor originating from the central cells of the pancreatic acinus, accounting for approximately 1% to 2% of all pancreatic exocrine tumors.^[7]^ Most SCN lesions are benign, with only rare cases progressing to serous cystadenocarcinoma.^[5]^ For typical SCN cases, especially in elderly and frail patients, nonsurgical observation of imaging characteristics can facilitate accurate diagnosis, which holds significant clinical value.^[12]^ The substantial variations in imaging presentations among the different types of SCN significantly contribute to misdiagnosis.^[13,14]^ Therefore, a comprehensive comparison of imaging findings, in conjunction with pathological insights, is essential to better delineate these imaging characteristics and improve diagnostic understanding.

SCN predominantly affects middle-aged and elderly women, with the average age of diagnosis being around 60 years.^[15]^ Most patients are asymptomatic, though some may experience abdominal discomfort or pain,^[7]^ which is consistent with the findings in our study. Our results showed no significant differences in age, location, or clinical symptoms among the various SCN types. Notably, the mixed type was observed to be larger than the microcystic type at initial detection, possibly due to variations in growth rates and detection rates, which warrants further investigation with larger sample sizes.

The differences in precontrast CT density among SCN types reflect their underlying pathological composition: the macrocystic type, characterized by large cystic cavities and sparse fibrous stroma, appears as watery low density; in contrast, the mixed and microcystic types, which contain abundant fibrous stroma and densely packed microcysts, show significantly higher density. This difference has practical clinical value – when a pancreatic cystic lesion shows precontrast CT density significantly higher than that of simple fluid, microcystic or mixed-type SCN should be prioritized over macrocystic type or other simple cystic neoplasms.

When combining imaging with pathology, we found that the degree of tumor enhancement correlated with the proportion of stromal components, with abundant stromal blood vessels contributing to the enhancement. Since the proportion of stromal components varied across SCN types, the degree of enhancement also varied. In our single case of the solid type, which had the highest proportion of stromal components, the most pronounced enhancement was observed, while the macrocystic type exhibited the least. The microcystic and mixed types typically displayed moderate to significant enhancement, reflecting their rich stromal vasculature.^[16]^ Furthermore, our single case of the solid type featured mature small blood vessels, explaining its conspicuous enhancement, with intact vascular endothelial cells reducing the likelihood of bleeding.^[17]^ However, it should be noted that these observations regarding the solid type are based on a single case and require validation in larger cohorts.

Variations in lumen size, fibrous stroma ratio, and vascular richness were observed among SCN types, and even within the same type. Therefore, the mixed and microcystic types may exhibit mild, moderate, or significant enhancement, limiting the utility of enhancement degree alone for SCN diagnosis. Additionally, different tumor regions exhibited distinct enhancement patterns, such as delayed enhancement or wash-in and washout patterns,^[16]^ reflecting the variable vascular densities. The presence of fibrous scars within lesions is diagnostically significant,^[11]^ as dense fibrous tissue typically shows delayed enhancement on imaging.

The distribution of fibrous scars shows a clear subtype dependence: they are common in microcystic and mixed types but completely absent in the macrocystic type. On T2WI, scars exhibit characteristic low signal intensity, sharply contrasting with the high signal of intracystic fluid – this is the key advantage of MRI.^[11,18]^ Accordingly, the following imaging strategy could be recommended: CT as the initial screening modality to detect calcification (a relatively specific sign of SCN, though with a low incidence of <30%); when CT findings are atypical or scar confirmation is needed, supplemental MRI may provide critical diagnostic information, particularly in confirming microcystic or mixed-type SCN and avoiding misdiagnosis as mucinous cystic neoplasm (MCN) or PanNET.

The reported incidence of calcification in SCN varies across existing studies (11.5–27.1%),^[5,7,8,13,19]^ which may be attributed to differences in total sample sizes and variations in the composition of pathological subtypes across studies. Our data showed that the incidence of calcification in microcystic and mixed types was higher than that in the macrocystic type, consistent with the findings of a multicenter study.^[7]^ The incidence of central fibrous scars in SCN also differs among pathological subtypes, with higher rates in microcystic and mixed types and complete absence in the macrocystic type, which is consistent with previous pathology-imaging correlation studies.^[20]^ The detection efficacy of SCN is closely related to the imaging modality used. In our study, the detection rate of central fibrous scars on MRI was significantly higher than that on CT (50.0% vs 13.2%). The possible reasons are as follows: MRI, particularly with its multi-sequence imaging capabilities, offers superior soft tissue resolution and a characteristic low-signal-to-high-background contrast of fibrous scars, significantly improving the identification of small scars; differences in CT contrast-enhanced scanning phase protocols (60.3% of cases in our study lacked delayed-phase data) may result in incomplete capture of the enhancement characteristics of fibrous components.

The overall misdiagnosis rate was 41.5% on CT and 31.3% on MRI, with misdiagnosis patterns showing clear subtype dependence. The macrocystic type was primarily confused with MCN,^[18,21]^ as both share overlapping imaging features (unilocular/paucilocular large cysts, absence of central scars). The mixed and solid types were mainly mistaken for PanNET due to the hypervascular enhancement pattern of their solid components.^[17,22]^ The microcystic type was confused with other cystic-solid tumors when tiny microcysts appeared relatively solid. These findings may have practical clinical implications: when encountering a unilocular large cystic lesion, MCN should not be diagnosed solely based on “female, body/tail location”; macrocystic SCN could be considered in the differential. When encountering a hypervascular solid small mass, PanNET should not be diagnosed solely based on enhancement pattern; solid-type SCN could be suspected, with careful evaluation for microcystic components or central scars.

Despite the variable presentations across subtypes, SCN shares several common imaging features, among which the lobulated contour is the most diagnostically valuable. This feature, which may appear as the “extracapsular cyst sign”^[20]^ is observed across all subtypes (macrocystic: 70.6–77.8%; mixed: 81.8–93.7%; microcystic: 58.6–86.4%) and may serve as an important distinguishing point from MCN (which typically appears round or oval). Furthermore, SCN typically does not communicate with the pancreatic duct; although larger lesions may compress the duct, invasive growth is absent, consistent with its benign biological behavior.^[23,24]^ The high T2WI signal of cystic fluid is useful for differentiating SCN from solid tumors. In clinical practice, when a lesion demonstrates lobulated contour, high T2WI signal, and no communication with the pancreatic duct, SCN could be suspected.

Highly vascularized SCN, as observed in our single solid-type case, often resembles PanNET, especially when lesions are small and CT primarily shows solid findings,^[25]^ leading to a high misdiagnosis rate. The unenhanced CT values of SCN are significantly lower than those of PanNET, and richly vascularized SCN shows a higher relative washout rate.^[22]^ Various imaging features – including tumor size, T2-weighted signal intensity, apparent diffusion coefficient values, and the presence of a fibrous capsule – may suggest solid-type SCN.^[22]^ Notably, MRI is superior in revealing central scars and fibrous septa within the lesion. Clinical history of endocrine disease, liver metastasis, and lymph node enlargement further aids in distinguishing PanNET from SCN.^[12]^

In daily radiology practice, a 4-step approach can be used to differentiate SCN from other pancreatic cystic tumors. First, assess the cystic architecture. Multiple microcysts with a honeycomb appearance may suggest SCN. A unilocular or paucilocular large cyst requires differentiation from MCN, especially in female patients with lesions located in the pancreatic body/tail and thick cyst walls.^[21,24]^ A mixed cystic-solid appearance warrants differentiation from solid pseudopapillary neoplasm (SPN) or PanNET. Multilocular cysts communicating with the pancreatic duct may suggest intraductal papillary mucinous neoplasm. Second, look for a central scar. The presence of a central fibrous scar strongly supports SCN (microcystic or mixed type), while its absence does not exclude other diagnoses. Third, evaluate the enhancement pattern. No significant enhancement may suggest macrocystic SCN or MCN. A hypervascular fast-in fast-out enhancement pattern requires differentiation from PanNET. Progressive enhancement may suggest SPN. Fourth, integrate clinical features. A young female patient with a typical “floating cloud sign” may supports SPN.^[3]^ The presence of endocrine symptoms and liver metastasis may support PanNET. An elderly male patient with pancreatic duct dilation may support intraductal papillary mucinous neoplasm.

Despite certain imaging differentials, SCN with atypical features poses diagnostic challenges. Radiomics and deep learning hold promise for improving the differential diagnosis of SCN.^[21,26,27]^ Recent studies demonstrate that radiomics-based approaches can perform comparably to experienced radiologists,^[28]^ although further validation is required. Endoscopic ultrasonography has diagnostic value through cyst fluid aspiration and fine-needle puncture biopsy,^[29]^ but its limited availability restricts its widespread use. CT and MRI remain the primary diagnostic modalities for SCN. Therefore, a comprehensive understanding of the differential diagnosis of pancreatic cystic tumors, as well as of SCN types, enhances diagnostic accuracy and may help avoid unnecessary surgeries.

This study has several limitations. First, the low incidence of SCN resulted in a small sample size, with only 1 solid-type case, limiting statistical power for subgroup analyses. Since the study only included pathologically confirmed cases, the study population may not fully represent the full range of SCN encountered in routine clinical practice. Second, the retrospective design and lack of delayed-phase imaging in some patients may have affected fibrous scar visualization. Third, imaging protocol heterogeneity: CT scans used 3 different scanners, which may potentially limit cross-device comparability of absolute CT values; MRI used both 1.5T and 3.0T scanners, and field strength differences may influence signal interpretation. The impact of this technical heterogeneity on the results is difficult to quantify. Fourth, this was a comparative analysis rather than a diagnostic accuracy study; no diagnostic performance metrics were calculated, and the conclusions should not be directly generalized to diagnostic settings. Future multicenter, prospective studies with standardized protocols are needed.

In conclusion, the imaging differences among SCN subtypes arise from variations in cyst size, fibrous stroma proportion, and vascular abundance. A comprehensive understanding of these features aids in imaging differentiation and clinical decision-making. The key clinical implications are as follows: fibrous scar is a key marker for subtype differentiation (present in microcystic and mixed types, absent in the macrocystic type); MRI is superior to CT in demonstrating microcystic architecture; and solid-type SCN may be prone to misdiagnosis as PanNET, requiring differentiation using precontrast CT values and enhancement indices.

## Author contributions

**Conceptualization:** Guangmang Li, Aichun Lei, Jianli Zhao, Xiao Han.

**Data curation:** Guangmang Li, Aichun Lei, Jianli Zhao, Peng Wang, Guanghai Ji.

**Formal analysis:** Guangmang Li, Peng Wang.

**Writing – original draft:** Guangmang Li, Aichun Lei, Jianli Zhao, Peng Wang, Guanghai Ji, Xiao Han, Peng Li, Bo Li.

**Writing – review & editing:** Guangmang Li, Aichun Lei, Jianli Zhao, Peng Wang, Guanghai Ji, Xiao Han, Peng Li, Bo Li.




