# Supplementary material for: A retrospective comparative study of CT and MRI findings of different types of pancreatic serous cystic neoplasms: Correlation with histopathology
Source: Medicine (Baltimore). 2026 Aug 21;105(34):e50307. doi: 10.1097/MD.0000000000050307 (PMC13502790; doi:10.1097/MD.0000000000050307)
Supplement: Supplementary file 1 [file medi-105-e50307-s001.docx]

**Supplemental Digital Content Table S1 Misdiagnosis of CT scans for different types of SCN**

| **Misdiagnosis of other tumors,n(%)** | **Macrocystic type(n＝17)** | **Mixed type(n＝11)** | **Microcapsule type(n＝24)** | **Solid type(n＝1)** | **Total (n**  **＝53)** |
| --- | --- | --- | --- | --- | --- |
| MCN | 4(23.5) | 0(0) | 0(0) | 0(0) | 4(7.5) |
| Pseudocyst | 2(11.8) | 0(0) | 0(0) | 0(0) | 2(3.8) |
| Retention cyst | 1(5.9) | 0(0) | 0(0) | 0(0) | 1(1.9) |
| PNET | 0(0) | 3(27.3) | 1(4.2) | 1(100.0) | 5(9.4) |
| SPN | 0(0) | 0(0) | 4(16.7) | 0(0) | 4(7.5) |
| PDAC | 0(0) | 0(0) | 1(4.2) | 0(0) | 1(1.9) |
| IPMN | 1(5.8) | 0(0) | 1(4.2) | 0(0) | 2(3.8) |
| Indeterminacy | 0(0) | 0(0) | 3(12.5) | 0(0) | 3(5.7) |
| Total | 8(47.1) | 3(27.3) | 10(41.6) | 1(100.0) | 22(41.5) |

SCN, pancreatic cystic neoplasm; MCN, Mucinous cystic neoplasm; PNET, Pancreatic neuroendocrine tumor; SPN, solid pseudopapillary neoplasm; PDAC, Pancreatic ductal adenocarcinoma; IPMN, intraductal papillary mucinous neoplasm.
