# Supplementary material for: A retrospective comparative study of CT and MRI findings of different types of pancreatic serous cystic neoplasms: Correlation with histopathology
Source: Medicine (Baltimore). 2026 Aug 21;105(34):e50307. doi: 10.1097/MD.0000000000050307 (PMC13502790; doi:10.1097/MD.0000000000050307)
Supplement: Supplementary file 2 [file medi-105-e50307-s002.docx]

**Supplemental Digital Content Table S2 Misdiagnosis of different types of SCN on MRI**

| **Misdiagnosis of other tumors, n (%)** | **Macrocystic type(n＝9)** | **Mixed type(n＝16)** | **Microcapsule type(n＝22)** | **Solid type(n＝1)** | **Total (n**  **＝48)** |
| --- | --- | --- | --- | --- | --- |
| MCN | 4(44.4) | 0(0) | 0(0) | 0(0) | 4(8.3) |
| Pseudocyst | 1(11.1) | 0(0) | 0(0) | 0(0) | 1(2.1) |
| PENT | 0(0) | 3(18.7) | 4(18.2) | 1(100.0) | 8(16.6) |
| IPMN | 0(0) | 0(0) | 1(4.5) | 0(0) | 1(2.1) |
| PDAC | 0(0) | 0(0) | 1(4.5) | 0(0) | 1(2.1) |
| Total | 5(55.5) | 3(18.7) | 6(27.3) | 1(100.0) | 15(31.3) |

SCN, pancreatic cystic neoplasm; MCN, Mucinous cystic neoplasm; PNET, Pancreatic neuroendocrine tumorPDAC, Pancreatic ductal adenocarcinoma; IPMN, intraductal papillary mucinous neoplasm.
